# Chemically Diverse and Biologically Active Secondary Metabolites from Marine *Phylum chlorophyta*

**DOI:** 10.3390/md18100493

**Published:** 2020-09-26

**Authors:** Sayed Asmat Ali Shah, Syed Shams ul Hassan, Simona Bungau, Yongsheng Si, Haiwei Xu, Md. Habibur Rahman, Tapan Behl, Daniela Gitea, Flavia-Maria Pavel, Raluca Anca Corb Aron, Bianca Pasca, Sebastian Nemeth

**Affiliations:** 1 Key Laboratory of Advanced Drug Preparation Technologies, Ministry of Education, Co-Innovation Center of Henan Province for New Drug R&D and Preclinical Safety, School of Pharmaceutical Sciences, Zhengzhou University, Zhengzhou 450001, China; sayedasmat89@yahoo.com (S.A.A.S.); siys01@126.com (Y.S.); 2 Shanghai Key Laboratory for Molecular Engineering of Chiral Drugs, School of Pharmacy, Shanghai Jiao Tong University, Shanghai 200240, China; Shams1327@yahoo.com; 3 Department of Natural Product Chemistry, School of Pharmacy, Shanghai Jiao Tong University, Shanghai 200240, China; 4 Department of Pharmacy, Faculty of Medicine and Pharmacy, University of Oradea, 410028 Oradea, Romania; gitea_daniela@yahoo.co.uk (D.G.); biancapasca28@yahoo.com (B.P.); sebinemeth@yahoo.com (S.N.); 5 Department of Pharmacy, Southeast University, Banani, Dhaka 1213, Bangladesh; pharmacisthabib@gmail.com; 6 Department of Pharmacology, Chitkara College of Pharmacy, Chitkara University, Punjab 140401, India; tapanbehl31@gmail.com; 7 Department of Preclinical Disciplines, Faculty of Medicine and Pharmacy, University of Oradea, 410073 Oradea, Romania; flavia.bontze@gmail.com (F.-M.P.); raluca14@yahoo.com (R.A.C.A.)

**Keywords:** chlorophytes, alkaloids, terpenes, steroids, fatty acid, glycerides, lipids

## Abstract

For a long time, algal chemistry from terrestrial to marine or freshwater bodies, especially chlorophytes, has fascinated numerous investigators to develop new drugs in the nutraceutical and pharmaceutical industries. As such, chlorophytes comprise a diverse structural class of secondary metabolites, having functional groups that are specific to a particular source. All bioactive compounds of chlorophyte are of great interest due to their supplemental/nutritional/pharmacological activities. In this review, a detailed description of the chemical diversity of compounds encompassing alkaloids, terpenes, steroids, fatty acids and glycerides, their subclasses and their structures are discussed. These promising natural products have efficiency in developing new drugs necessary in the treatment of various deadly pathologies (cancer, HIV, SARS-CoV-2, several inflammations, etc.). Marine chlorophyte, therefore, is portrayed as a pivotal treasure in the case of drugs having marine provenience. It is a domain of research expected to probe novel pharmaceutically or nutraceutically important secondary metabolites resulting from marine *Chlorophyta*. In this regard, our review aims to compile the isolated secondary metabolites having diverse chemical structures from chlorophytes (like *Caulerpa* ssp., *Ulva* ssp., *Tydemania* ssp., *Penicillus* ssp., *Codium* ssp., *Capsosiphon* ssp., *Avrainvillea* ssp.), their biological properties, applications and possible mode of action.

## 1. Introduction

Developed throughout the ages to code biological processes, the products of nature are the predecessors of medicines and the dawning of drugs. Previously just the craving to cure illness, conventional remedies and potions ceded substantial territory towards those considered more competent modern chemical techniques for developing new medicines [1]. However, the chemotypes designated by natural components are amid the highly adroit for encompassing the spectrum of pathogens that continue to affect significant human population health, being an economic burden worldwide. For example, the antimicrobial beta-lactams emerged by fungus-like *Penicillium* sp., and the quinine alkaloid obtained from the bark of cinchona tree lodge are prototypical examples of esteemed therapeutically active substances with fringe benefits.

Products of natural origin used to be the nucleus of the pharmaceutical armamentarium, ready-to-access compared to synthetical products and decreased concern in maintaining an archetype of discovery featuring fermentation, isolation, structural establishing and biological assay of the new natural pharmacologically active compounds [2]. Nevertheless, the synthetic/semisynthetic substances and/or natural products, influenced or natural-product-based, even the natural products themselves have proved to be a treasure with a substantial potential and contribution to today’s drugs [3,4]; also, they retain a valuable tool for the amplification of the pharmacology to the target space for emerging diseases [5,6].

Research concern has greatly enhanced as regards the marine life, marine algae and their amazing secondary metabolites started to be used for their strong therapeutic activities in the last decades [7,8]. According to recent studies, about 72,500 species of algae throughout the world have been determined, whereas marine algae are at high peak among them, representing a large group among the marine organisms [9]. The algae are brown (*Phaeophyta*), red (*Rhodophyta*) and green (*Chlorophyta*).

Marine algae are influenced by environmental parameters such as climate, salinity, pH, sunlight, physiological conditions and CO_2_ supply [10,11]. Because of different adaptation strategies, macroalgae can survive in harsh environmental conditions. Due to the adaptive nature of the macroalgae’s physiology, they may produce numerous secondary metabolites with exclusive structural cores encompassing alkaloids, cyclic peptides, diterpenoids, glycerol, lipids, phlorotannin, polyketides, polysaccharides, quinones and sterols [7,8,12]. Chlorophytian seaweeds are abundant in intertidal and deep-water areas of the seas, popularly known as green algae.

Secondary metabolites resulting from *Chlorophyta* can establish the podium for the synthesis of inventive healing drugs, being efficient to combat a variety of tolerant pathogens [13]. In fact, the genus *Caulerpa* is relevant for numerous of the green-algae-derived anticancer compounds recounted to date [14].

**Figure 1:**
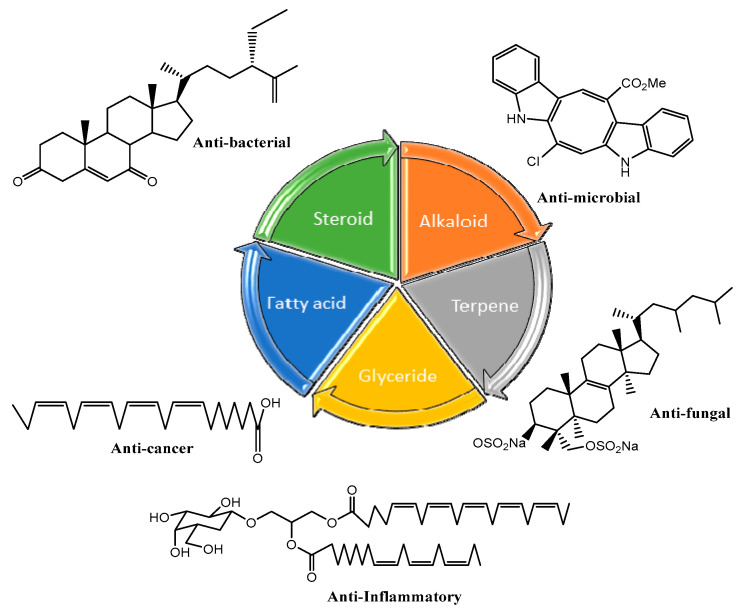
Structural diversity of compounds produced by *Chlorophyta*.

The main purpose of the present review-type paper is to highlight the chlorophyte-derived bioactive molecules that embodied in vivo potency or staggering in vitro activity vs. different kinds of tumors, parasitic, infectious diseases, etc., and their way of action through which they interpose with human pathogenesis. The review was classified according to the structural diversity of compounds produced by *Chlorophyta* and their biological spectrum, as presented in Figure 1.

## 2. Methods

A literature search was done in several databases including PubMed, Web of Science, Scifinder, Scopus, Google Scholar, Springer, Science Direct, Taylor and Francis, Bentham Sciences, American Chemical Society, MDPI and Wiley to identify reports published on “green algae/*Chlorophyta*” related to natural products isolation or pharmacological activities of compounds or extracts. Initially, all the literature regarding *Chlorophyta* was downloaded and then further classified according to its chemical diversity (alkaloids, terpenes, polysaccharides, etc.) and pharmacological potencies (antibacterial, anticancer, nutraceuticals, etc.). To the best of our knowledge, this study is the first classifying *Chlorophyta* according to its chemical diversity.

## 3. Green Algae, a Source of Bioactive Secondary Metabolites

According to recent research, about 72,500 species of algae have been identified worldwide, although most of them are marine, representing a significant community among marine organisms [9]. Recently, approximately 3200 natural products from marine macroalgae have been documented, representing 13% of compounds from marine organisms [15]. A broad variety of substances, especially terpenes, polyphenols and steroids have been identified in various marine green algae [16], within them a large part is constituted by terpenoid compounds. For instance, *Caulerpa brownii* found in Australia has been shown to contain various bioactive terpenoid esters and novel diterpenoids [17]. The anti-inflammatory agent was identified as 3-*O*-b-glucopyranosylstigmasta-5, 25-diene produced by *Ulva lactuca* [18]. Green algae of the *Caulerp*a genus is the primary source from which the alkaloid is mostly isolated, especially bisindole alkaloid, caulerpin or caulerpine [19], that has shown multiple bioactivities (antibacterial [20], antitumoral [14], inhibitor of human protein tyrosine phosphatase-1B (hPTP1B) [21] or of hypoxia-inducible factor (HIF)) [22], antiviral [23], antinociceptive and anti-inflammatory [24]. In short, green algae are the main source of diverse chemical structural bioactive secondary metabolites, having a broad range of pharmacological properties.

### 3.1. Alkaloids from Green Algae

According to Pelletier (1983), alkaloids are “cyclic organic compounds containing nitrogen in a negative oxidation state which are of a limited distribution among living organisms” [25]. In several groups of secondary metabolites, alkaloids are represented by large and highly structural compounds. The biological activity is conferred by the nitrogen found in the molecular structure, in the case of a great number of compounds from this class. The alkaloids produced by green algae chlorophyte are discussed below.

#### 3.1.1. Bisindole Alkaloids

Indole alkaloids, synthesized by a large variety of organisms, from marine and terrestrial environments, include numerous secondary metabolites with differing structures. In the marine habitat, many unique bisindole alkaloids are produced, green algae (*Chlorophyta*) being one of the main sources of producing bisindole alkaloids. Two novel bisindole alkaloids, racemosins A (**1**) (a pink amorphous powder) and B (**2**) (an orange-yellow amorphous powder), along with one well-known pigment caulerpin (**3**) were isolated from the green alga *Caulerpa racemosa* [26], and the sample was collected from the Zhanjiang coastline in the East China Sea, China. Racemosin A (**1**) is a bisindole alkaloid unique from the structural point of view, having a seco-indolo [3,2-a] carbazole skeleton and two uncommon indolinone parts mixed with a half of methyl propanoate and racemosin B its uncommon cyclic derivative (**2**). The indolinone system makes the racemosin A (**1**) structure distinctive, being found in some plant-based natural products and several synthetic products Furthermore, a review of the literature points out that indolo [3,2-a] carbazoles, were obtained through synthesis, ancorinazole being the only indolo [3,2-a] carbazole that occurs naturally being isolated from the sponge *Ancorina* sp. in New Zealand. Racemosin A (**1**), as well as racemosin B (**2**), showed neuroprotective action for Aβ_25–35_-induced SH-SY5Y cell damage, increasing cell viability with 14.6%, at a concentration of 10 μM as positive control when compared with epigallocatechin gallate (EGCG, 16.57% growth at 10 μM), while compound (**2**) exhibits moderate behavior. Yang and his team identified a novel small bisindole alkaloid, racemosin C (**3**), naturally unmatched 8-hydroxy-2,4,6-cyclo-octatrienone ring combined with two indole systems, along with caulersin (**2**), a well-known associated metabolite from the *Caulerpa racemosa* green alga [27]. Compounds like racemosin C (**3**) and caulersin **2** demonstrated PTP1B significant inhibitory activity with IC_50_ values of 5.86 ± 0.57 and 7.14 ± 1.00 µM, respectively. Caulerpin (**4**) and caulerpic acid (**5**), were defined in 2014 [28] as being bisindolic compounds, previously isolated from samples collected along the south Indian coasts and described based on literature data. Recently, samples collected from the South-East China Sea showed antinociceptive and anti-inflammatory activities; moreover, it was found that caulerpin (**4**) presents high PTP1B inhibitory activity with an IC_50_ value of 3.77 µM. Caulerpin (**4**) has an additional eight-member ring between the two indole rings, integrated into the carbonyl group; its nonselective spasmolytic action is partly determined by the inhibition of Ca^2+^ influx by voltage-gated calcium channels (Cav) [29]. Important green algae contain caulerpin (**4**) (i.e., *Caulerpa racemosa, C. sertularioides, C. serrulata, C. lamourouxii* and many other species of *Chlorophyta*); it was also revealed that caulersin (**6**), an abisindole alkaloid, has two “antiparallel” indole nuclei, isolated from *Caulerpa serrulata* sp. [30]. Caulerchlorin (**7**), showing low antifungal activity against *Cryptococcus neoformans* 32609 strain (MIC_80_ 16 μg/mL), was isolated from *Caulerpa racemosa* [31]. Caulerpin (determined from the algae lipoid extract *Caulerpa racemose*) demonstrated anti-inflammatory behavior in mice when administered orally at a concentration level of 100 μmol/kg; also, its bisindolic pharmacophoric nucleus is most likely responsible for the broad range of reported biological properties, such as being an anti-inflammatory agent and antinociceptive [32].

##### Mode of Action

Pharmacologically active substances contain alkaloids derived from natural sources used as anti-inflammatory, antimicrobial and antifungal medicines. Inflammation is part of the body’s immune response. The immune system identifies irritants, parasites and damaged cells and the healing process is initiated. The symptoms of inflammation include discomfort, redness and swelling. Inflammation is considered also the self-protective mechanism of the body, in the purpose of eliminating injurious stimuli and to begin the healing process.

The inflammatory response starts with the identification of an inflammatory or infectious origin signal and the release of chemicals from migrating cells and tissues called mediators [33]. These mediators were identified as amines such as 5-hydroxytryptamine and histamine, bradykinin, interleukin-1 (IL-1) and lipids such as prostaglandins (PGs). The inflammatory cycle is the body response to an antigen, infectious agent or damage of exposed cells. Inflammation is the most frequent symptom of the disease and it is a pathophysiological mechanism involving complex pathways that are often triggered by bacterial degradation products from different microorganisms; lipopolysaccharides, lipopeptides, formyl methionyl peptides peptidoglycans, viruses (double-stranded RNA), fungi (zymosans) or even the body’s cells after damage and death [34]. Anti-inflammatory drugs achieve their therapeutic effects by receptor activation and enzyme inhibition. Currently, the major pathophysiological pathways for drug targeting are metabolism of arachidonic acids; phagocytosis; autoimmune processes; the cascade complement and other cell functions; protein kinase C and other enzymes involved in second messenger systems [1,5]. Among anti-inflammatory and analgesic drugs, alkaloids continue to be effective therapeutic agents for chronic and severe pain [35]. The alkaloid pharmacological studies have focused on the alkaloid therapeutic effects implied in the physiological processes.

**Figure 2:**
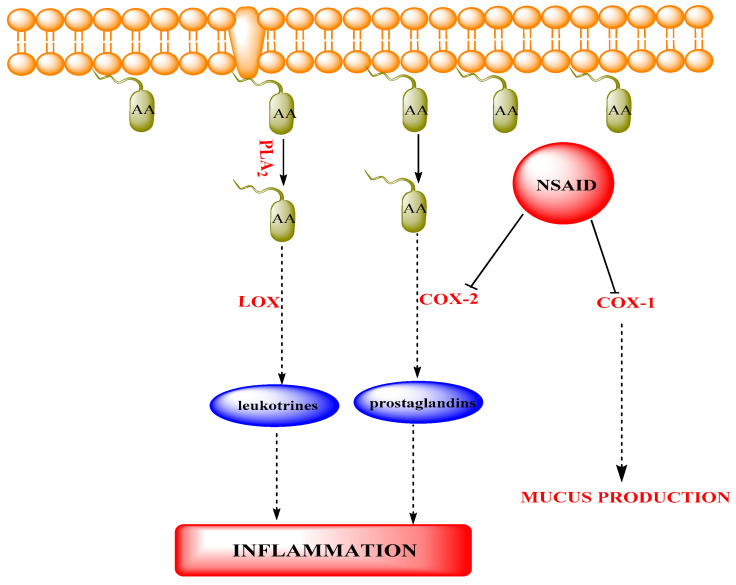
Diagrammatic process of inflammatory cascade inside the cell. Phospholipase A2 (PLA2) catalyzes the release of membrane-bound arachidonic acid (AA) to free arachidonic acid. Arachidonic acid is then converted to leukotrienes and prostaglandins by lipoxygenase (LOX) and cyclooxygenase-2 (COX-2), respectively. Alkaloidal substances inhibit inflammation by different targets mentioned in the cascade.

Several isoquinoline alkaloids (berberine, berbamine and cepharanthine) were tested for their anti-inflammatory action [36]. Anti-inflammatory drugs function, in basic terms, by reducing inflammation. By antagonizing the main inflammation enzyme called cyclooxygenase (COX), which transforms arachidonic acid into leukotrienes and prostaglandins, they reduce inflammation. The responsibility for local inflammation lies with prostaglandins. Hence, anti-inflammatory drugs reduce inflammation by blocking cyclooxygenase [37]. Recent evidence indicates the existence of two different variants of the cyclo-oxygenase enzyme, namely COX-1/ COX-2. COX-1 is a constituent part of normal cells and COX-2 is produced in inflamed cells. The most likely mechanism of action for nonsteroidal anti-inflammatory drugs (NSAID-mediated analgesia) is a COX-2 inhibitory activity [38], briefly described in Figure 2.

#### 3.1.2. Other Alkaloids and Prenylated Compounds

Further analysis of the same species (*Caulerpa racemosa*) led Liu [26] to identify two new prenylated para-xylenes collected from the coastline of Zhanjiang, China, called caulerprenylol A (**8**) and caulerprenylol B (**9**). This is the first research on marine algae and marine organism prenylated para-xylene. In addition, caulerprenylol B also exhibits an uncommon indane ring system; also present in a diversity of products obtained through synthesis, like indanoestrols A and B. Caulerprenylol B (**9**) displayed moderate antimycotic activity towards *Candida glabrata* (537), *Trichophyton rubrum* (Cmccftla) and *Cryptococcus neoformans* (32609), with 4.0, 16.0 and 4.0 µg/mL MIC_80_ value, respectively, while caulerprenylol A (**8**) showed weak antifungal activity with the same MIC_80_ value of 64.0 µg/mL against the above fungal strains [26]. Additionally, they were published data [39] on a new alkaloid that had an additional effect on human enzyme action. Pyrrolopiperazine-2,5-dione alkaloid (**10**), from the green algae *Ulva prolifera*, possesses antialgal activity against the frequent hazardous of red tide microalgae [40]. The secondary metabolites from *Cymopolia barbata*, composed of 7-Hydroxycymopochromanone (PBQI) (**11**), 7-Hydroxycymopolone (PBQ2) (**12**), prenylated bromo-hydro-quinones that are (′-methoxy-7-hydroxycymopolone (**13**), 3-hydroxycymopolone (**14**), 3,7-hydroxycymopolone (**15**), 7-hydroxycymopochromanone and 7-dihydroxycymopochromenol (**16**)-related 6-hydroxy derivatives of cymopochromenol (**17**), along with other like 1,4-dihydroxybenzene, dibromo-cymopolone, cymobarbatol and 4-isocymobarbatol. Both (PBQI) and (PBQ2) are reported to be chemotherapeutic compounds but make residue of the hydroxyl moiety (PB2) to act selectively against cancer colon cells. Crude extracts of *Cymopolia barbata* blocked progesterone-stimulated gene expression in human progesterone receptor cells and purified cyclic epimeric bromo-hydro-quinones (cymobarbatol and 4-isocymobarbatol), showing antimutagenic activity against *Salmonella typhimurium* [41,42].

**Figure 3:**
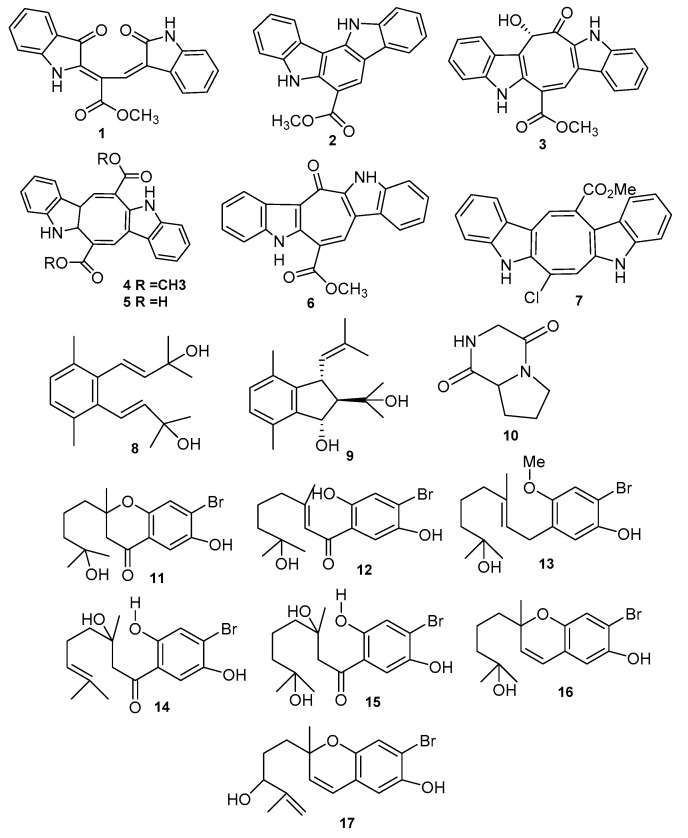
The names of Compounds **1**–**17** are represented in Table 1.

The chemical structures of alkaloids and prenylated compounds from *Chlorophyta* are presented in Figure 3 and the species and their bioactivity are described in Table 1.

### 3.2. Terpenes from Green Algae

Terpenoids are recognized as a large category of secondary metabolites having a vast structural and functional variety, constructed by isoprene units. Terpenoids may be categorized (mono-, sesqui-, di-, sester-, tri- and tetraterpenoids), based on the number of isoprene units [41]. Additionally, the carotenoids can be classified under terpenoids, as being tetraterpenoid derivatives that have eight isoprene units [43].

#### 3.2.1. Sesquiterpenes

Three units of isoprene, the backbone of the C15 carbon is called sesquiterpene. Two new sesquiterpenes, with an unusual aromatic carbon skeleton of valerenane type (caulerpal A (**18**) and B (**19**)), were isolated from the Chinese green alga *Caulerpa taxifolia* [21], along with one known metabolite, caulerpin. Both the compounds caulerpal A (**18**) and caulerpal B (**19**) show weaker activity against hPTP1B (human protein tyrosine phosphatase 1B) than caulerpin (**4**) with an IC_50_ value of 3.77 µM but they did not reveal any significant bioactivity towards HL-60 and MCF-7 cell lines.

#### 3.2.2. Mode of Action of PTPs Inhibitor

Protein tyrosine phosphatases (PTPs) are those enzymes that are supposed to control the response of the cell to external stimuli. The remarkable variety of these phosphatases (including more than 100 members) recognized in the human genome expresses the crucial role of these proteins performed in the routine biochemical practices. The anomalous role of individual phosphatases was accompanied by the pathogenesis of the inclusive variability of inherited or acquired human diseases [44].

Additional biochemical confirmation of PTP1B therapeutically targeting obesity and diabetes has been obtained from a diversity of sources, including overexpression in vitro, antisense oligonucleotide studies, mutation observation in the PTP1B human gene sequence and human single nucleotide polymorphisms [45]. Based on the previously mentioned information, PTP1B is perceived as one of the best confirmed biological targets for obese patients and non-insulin-dependent diabetes. Additionally, several groups have a well-known role for PTP1B in cancer [46,47]. For example, one of the demonstrations from Tremblay that PTP1B overexpression is adequate to initiate tumorigenesis in mice, supply further evidence in using PTP1B inhibitors in cancer therapy [48].

At present, PTP1B is being studied as a therapeutic target class. In human clinical trials, only no competitive small-molecule protein tyrosine phosphatase inhibitors are currently being identified. It was documented that the effect of algal products caulerpal A and B, which have structural similarity with caulerpin, has been studied and its role to inhibit hPTPs1B was reported in 2006. Caulerpin has been reported to possess inhibitory function for hPTPs1B and remarkable anticancer activity [14,21]. Additionally, it has been established the mechanism by which cancer can be suppressed by the inhibition of PTPs, in 2017. The Src homology 2 (SH2) domain composed of protein tyrosine phosphatase 1 (SHP-1) inhibits the signal activator of the transcription 3 signaling pathway transducer.

In cancer cells, phosphorylates signal activator and transducer of transcription 3 signaling pathways are activated by Janus-associated kinase (JAKs) [49]. This results in the following actions/effects: the translocation of active signal transducer and transcription activator 3 (p-STAT3) dimers to the nucleus; the activation of the 3-regulated cell proliferation, survival signal activator and transcription transducer, metastasis and angiogenesis of the endothelial growth factor (VEGF).

**Figure 4:**
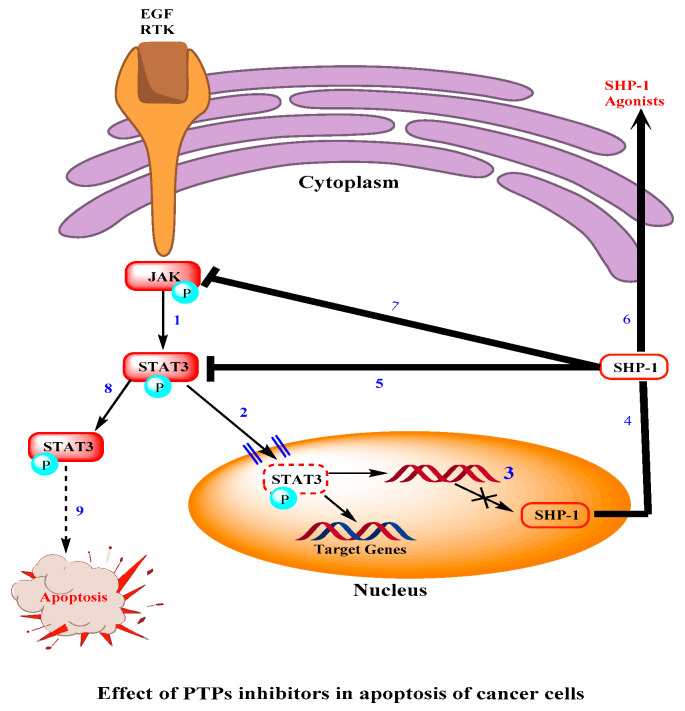
In cancerous cells, STAT3 phosphorylates by JAKs (1). Activated STAT3 moves towards the cell’s nucleus (2) and prevents the transcription of SHP1 (3) which results in the reduction of protein level (4). Overexpressing SHP1 via some of its agonists (7) results in the dephosphorylation of STAT3 (6) and also JAKs (8) to reduce the protein level (9). That results in the apoptosis of the cancer cell (10). Abbreviations: Extracellular growth factor (EGF), receptor tyrosine kinase (RTK), Src homology domain-containing protein tyrosine phosphatase (SHP-1), Janus-associated kinase (JAK), signal transducer and activator of transcription 3 (STAT3).

The activated STAT3 also forms, in the promoter region of the SHP-1 gene, complexes with deoxynucleic acid (DNA) methyltransferase 1 (DNMT1) to suppress its transcription, resulting in a decline in the protein level. Improved SHP-1 activity by SHP-1 agonists directly dephosphorylates STAT3 or its upstream JAKs to decline the p-STAT3 proteins supplemented by the blocking of cellular signaling pathways STAT3-mediated, as it is shown in Figure 4 [50,51].

**Figure 5:**
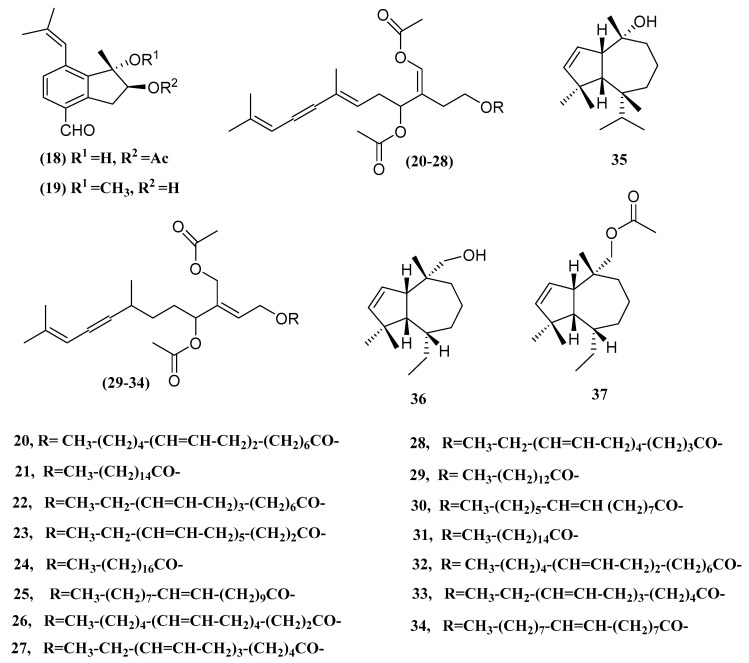
The names of compounds are given as **20**–**34** in Table 2.

In 2003, Smyrniotopoulos reported sixteen secondary metabolites of green algae *Caulerpa prolifera* known as acetylene sesquiterpenoid esters, having structural similarity to caulerpenyne which was isolated from several *caulerpa* organisms (*prolifera, racemosa, taxifolia* and *lanuginosa*). Two metabolite groups were identified: 1,2-dihydro-(**20**–**28**) and 1,2,3,3′tetrahydro-2,3-didehydro (**29**–**34**), caulerpenyne carbon backbone, have been recognized. The caulerpenyne terminal vinyl acetoxy group was replaced by various residues of fatty acids. The first group of metabolites isolated consisted of nine esters (**20**–**28**), sharing the same 1,2-dihydro caulerpenyne (**2**) sesquiterpene skeleton, previously described. The second group of compounds (**29**–**34**) is characterized by 1,2,3,3′-tetrahydro-2,3-didehydro caulerpenyne (**3**), a sesquiterpenoid moiety previously identified only as C aldehyde from taxifolia. The resistance to bacteria *C. prolifera* extract was measured against six nonidentified marine bacterial strains. Two Gram-positive bacteria and one Gram-negative marine bacterium were moderately affected by the extract [52]. Chakraborty isolated two new guaiane sesquiterpene derivatives from green algae *Ulva fasciata*, guai-2-en-10α-ol (**35**) and guai-2-en-10α-methanol (**36**). Acetylation of 2 at position C11 gave guai-2-en-10α-methyl methanoate (**37**) with acetyl group [53]. The Compounds **2** and **3** demonstrated significant inhibition of Vibrio parahaemolyticus growth with minimal inhibitory concentrations of 25 and 35 µg/mL [53]. Chemical structures of sesquiterpene from *Chlorophyta* are presented in Table 2 and Figure 5 describes the species and their bioactivity.

### 3.3. Diterpenoids

Four units of isoprene, the backbone of C20, carbon are called diterpene. Seven labdane diterpenoids (**38**–**44**) namely labda-14-ene-8-ol (**38**), labda-14-ene-3α,8α-diol (**39**), labda-14-ene-8α,9α-diol (**40**) labda-14-ene-8α-hydroxy-3-one (**41**), ent-labda-13 (**16**),14-diene-2-one (**42**), ent-labda-13(16),14-diene-3α-ol (**43**), ent-labda-13 (**16**) and 14-diene-3α-ol (**44**), were presented by Chakraborty [53] as chief components, from green alga *Ulva fasciata* [54]. Labdane derivatives (**38**–**41**) were greater than labdane derivatives (**42**–**44**) as antibacterial assay against three fish pathogenic bacteria, namely *V. parahaemolyticus* MTCC 451, *V. vulnificus* MTCC 1145 and *Vibrio alginolyticus* MTCC 4439. The two diterpenoids, labda-14-ene-3α,8α-diol (**39**) and labda-14-ene-8α-hydroxy-3-one (**41**), inhibited the growth of *Vibrio parahaemolyticus* and *Vibrio alginolyticus* with the lowest amount of inhibitory concentrations of 30 µg/mL by (**39**), and 40 µg/mL by (**41**), respectively. Most of the sesquiterpene compounds were isolated from different Caulerpa species having feeding preference, antimicrobial, ichthyo-toxicity and feeding deterrents [55].

Three diterpenoids were extracted from *C. racemosa* along with 12 known compounds as well as a pair of epimers (racemobutenolids A and B (**45ab**)), 4,5-dehydrodiodictyonema A (**46**), an α-tocopheroid (α-tocoxylenoxy (**47**)), and a 28-oxostigmastane steroid. The epimers (**45ab**) are two unexpected diterpenoid lactones bearing a moiety of β*-*methyl-γ-substituted butenolide, whereas **46** and **47** symbolize the first natural products that have an ester group of hematinic acid and 3,5-dimethyl phenoxy functionality. Compounds **46** and **47** revealed a preventive activity against PTP1B, but Compound **47** verified strong inhibitory activities against PTP1B with 2.30 µM of IC_50_ values [56].

**Figure 6:**
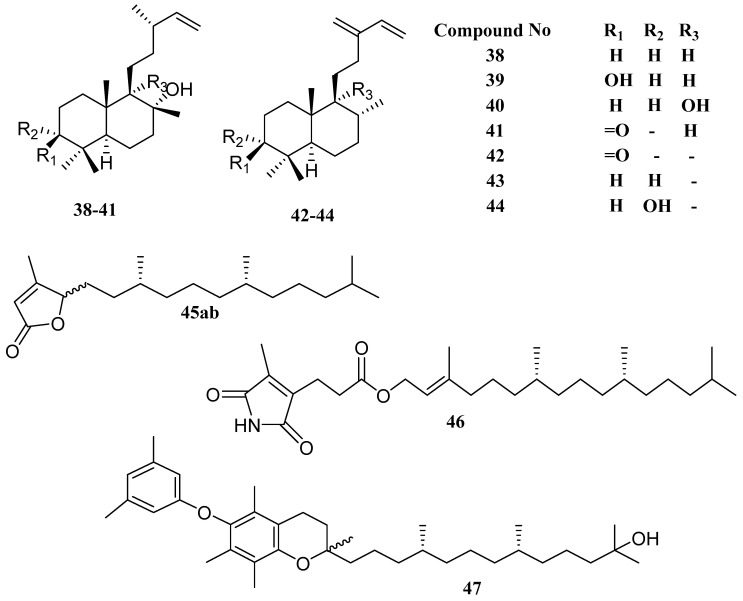
The names of compounds are represented as **38**–**47** in Table 3.

The chemical structures of diterpenoids from *Chlorophyta* are presented in Figure 6 and Table 3 presents the species and their bioactivity.

### 3.4. Triterpenoids

Four sulfate-conjugated triterpenoids were revealed, as follows: one new lanostane-type triterpenoid disulfate (lanosta-8-en-3,29-diol-23-oxo-3,29-disodium sulfate (**48**)) and three well-known cycloartane-type triterpenoid disulfates (cycloartan-3,29-diol-23-one 3,29-disodium sulfate (**2**), cycloart-24-en-3,29-diol-23-one 3,29-disodium sulfate (**3**) and cycloartan 3,23,29-triol 3,29-disodium sulfate (**4**)), from green macroalga *Tydemania expeditionis* [57]. Compound (**48**) was determined by comparing it to absolute configurations of (**2**–**4**), the configuration previously assigned at C-5. Disulfated natural products (**48** and **2**–**4**) exhibited weaker antitumor effects ranging from 31 to 38 μM, with IC_50_ values. That natural product has been moderately cytotoxic in toxicity assays for tumor cells in invertebrates. Among the natural products on view, only (**4**) demonstrated substantial antifungal activity against marine pathogen *Lindra thalassiae*, at natural concentrations of whole tissue. In 2003, Puglisi [58] isolated from the tropical green alga *Penicillus capitatus* two new triterpene sulfate esters, capisterones A (**49**) and B (**50**), found in the Tropical Atlantic Ocean. Such compounds are potent antifungal compounds which can protect *P. capitatus* against the indiscriminate marine pathogen *L. thallasiae*. This fungus’ growth was inhibited at concentrations of 0.03 and 0.94 mg/mL, in LD_50_. Squalene (**51**), which together with α-tocopherol, is a regular, special triterpene, isolated from *C. Racemosa* by Ragasa from Roxas City Philippines [59]. Ali, in 2015, identified more compounds: a novel triterpenic acid; dwarkenoic acid (**52**) and the known sterols; androst-5-en-3β-ol (**2**), stigmasta-5,25-dien-3β,7α-diol (**3**), ergosta-5,25-dien-3β-ol (**4**), 7-hydroxystigmasta-4,25-dien-3-one-7-*O*-β-d-fucopyranoside (**5**), 7-hydroxystigmasta-4,25-dien-3-one (**6**) and stigmasta-5,25-dien-3β-ol (**7**), *Codium dwarkense* marine macro algae. Compound (**52**) showed significant inhibition (at all concentrations) for enzymatic alpha-glucosidase, while the dose-dependent response was shown by Compounds **2**, **3**, **5** and **7** while Compounds **4**–**6** showed moderate inhibition [60]. As well, two terpenoids (loliolide (**53**) and lsololiolide (**54**)) were isolated from green algae *Ulva prolifera* [61].

**Figure 7:**
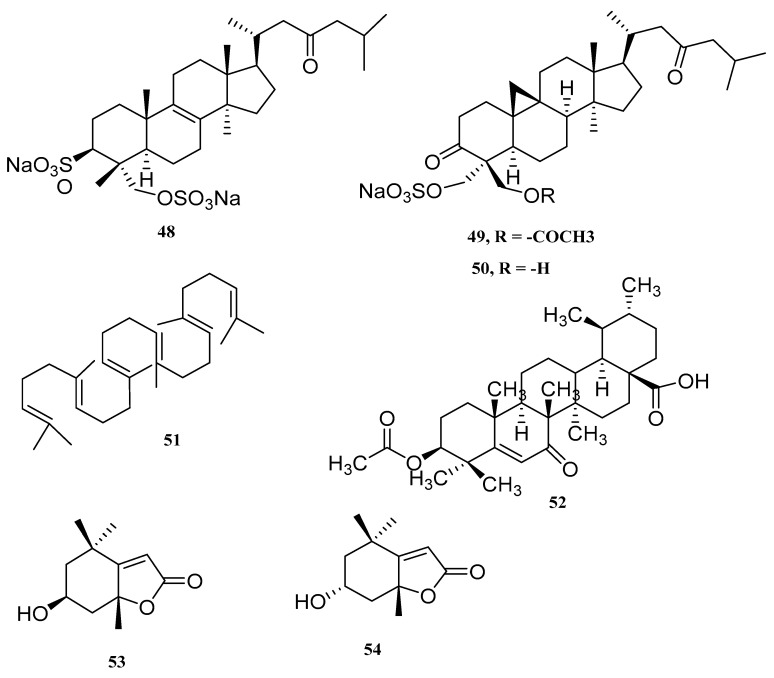
The names of compounds are represented as **48**–**54** in Table 4.

The chemical structures of triterpenoids from *Chlorophyta* are presented in Figure 7 and in Table 4 are depicted the species and their bioactivity.

### 3.5. Steroids and Fatty Acid

Sterols are triterpenoids compounds, consisting of **6** isoprene units. Fucosterol is the predominantly encountered sterol in brown algae, having different bioactivities. Red algae are rich in cholesterol, while green algae contain a variety of steroids [62]. Of the recognized anti-inflammatory drugs group, steroidal compounds and glucocorticoids, for instance, have the most powerful anti-inflammatory activity compared to the nonsteroidal anti-inflammatory drugs. These molecules have a somewhat different mechanism of action, while they first block eicosanoid growth by suppressing phospholipase A2 with lipocortin-1 synthesis [32].

A novel sterol: cholest-5-en-3-ol (**55**) isolated from green alga *Ulva prolifera* has potent antialgal activity against the typical hazardous red tide microalgae [61]. Mao [63] showed that new polyacetylenic fatty acid, (8*E*,12*Z*,15*Z*)-10-hydroxy-8,12,15-octadecatrien-4,6-diynoic acid (**56**); five known metabolites, including two linear diterpenes (**3** and **4**); and three sterols (**5**–**7**) were confirmed in the Chinese green algae *Caulerpa racemosa*. Trans-phytyl acetate (**4**) was first isolated from this species, before it was previously isolated from the green algae, *Ulva pertusa* [64].

Alamsjah [64] isolated three polyunsaturated fatty acids (PUFAs) from the green algae *Ulva fasciata*, known as:

- Hexadeca-4,7,10,13-tetraenoic acid (HDTA) (**57**);
- Octadeca-6,9,12,15-tetraenoic acid (ODTA) (**58**);
- α-linolenic acid (**59**), with strong algicidal activity against *Heterosigma akashiwo* (LC_50_ 1.35, 0.83 and 1.13 µg/mL for HDTA, ODTA and α-linolenic acid, respectively). It has to be mentioned that α-linolenic acid was isolated before and β-sitosterol (**60**) was isolated from *Caulerpa racemosa* [59].

Ali, in 2002, isolated a new steroid (**61**) (1, iyengadione), antibacterial steroidal glycosides and two new steroidal glycosides (iyengaroside A (**62**) and B (**63**)), along with clerosterol galactoside (**64**) from the marine green algae *Codium iyengarii* collected from Karachi coast. Compound **62** and **63** are active against *Klebsiella pneumonia* (with IC_50_ value 5.26 and 7.14 mg/mL, respectively), while Compound **61** activities have not been mentioned by the author [65].

Two novel unsaturated fatty acids, 3-hydroxy-octadeca-4(*E*),6(*Z*),15(*Z*)-trienoic acid (**65**) and 3-hydroxyhexadeca-4(*E*),6(*Z*)-dienoic acid (**66**), along with the known 3-hydroxy-octadeca-4(*E*),6(*Z*)-dienoic acid (**4**), were obtained from green alga *Tydemania expeditionis* [57]. Compounds **65**, **66** and **4** containing double conjugated bonds demonstrated mild inhibitory activity against a panel of tumor cell lines (breast, colon, lung, prostate and ovarian cells), with values of IC_50_ varying from 1.3 to 14.4 µM. In 2017, Li [66] isolated three new sterols, s(24*R*)-5,28-stigmastadiene-3β,24-diol-7-one (**67**), (24*S*)-5,28-stigmastadiene-3β,24-diol-7-one (**68**) and 24*R* and 24*S*-vinylcholesta-3β,5α,6β,24-tetraol (**69**), along with three recognized sterols (**4**–**6**) [66], obtained from green *alga Ulva australis*. For the first time, they were isolated Compounds **4**–**6** from *U. australis*. Compound **67** values (3.31 ± 0.850), **68** (4.08 ± 0.39) and **69** (2.87 ± 0.62) values have inhibitory effects on human recombining in vitro aldose reductase. Three mono-unsaturated fatty acid (MUFA) derivatives in the form of active substances were isolated from the green alga *Ulva lactuca* [67]**, comprising a new keto-type C18 fatty acid (**70**), the equivalent shorter chain C16 acid (**71**), and an amide derivative (**72**). These fatty compounds have a common structural feature, the conjugated α,β-unsaturated enone motif, which in the case of cysteine residues, may alkylate reactive thiol groups. The Michael acceptor motif is a regular denominator of many ARE activators, as stated earlier under tests. In 2014, Yang and his team [56], isolated 28-oxostigmastic steroid, (23*E*)-3b-hydroxy-stigmasta-5,23 dien-28-one (**73**), green algae *Caulerpa racemosa* having most active PTP1B inhibitory properties too, with an IC_50_ value of 3.80 µM.

**Figure 8:**
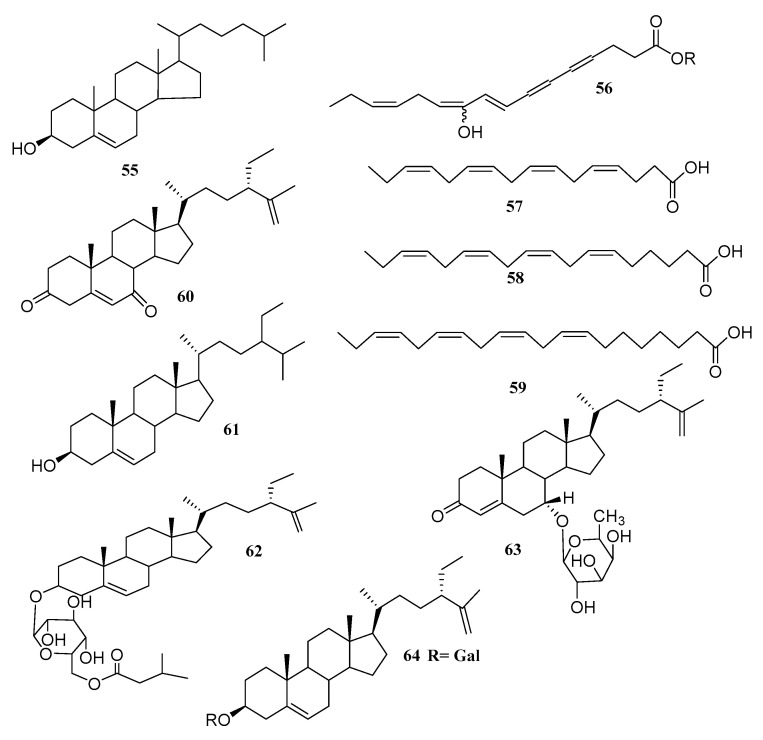

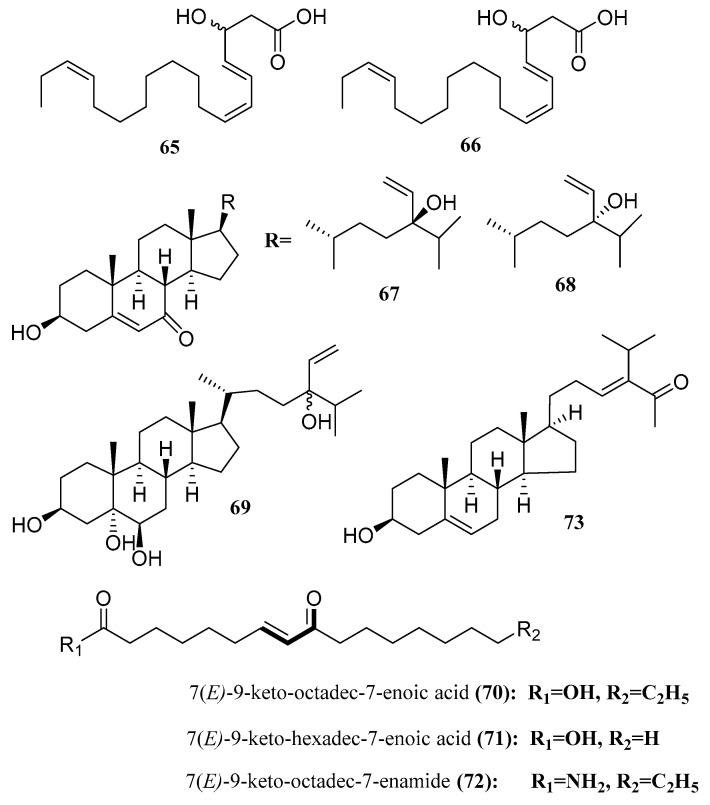
The names of compounds are denoted as **55**–**73** in Table 5.

The chemical structures of steroids and fatty acid from *Chlorophyta* are presented in Figure 8 and Table 5 describes the species and their bioactivity.

### 3.6. Glycerol and Lipids

**Figure 9:**
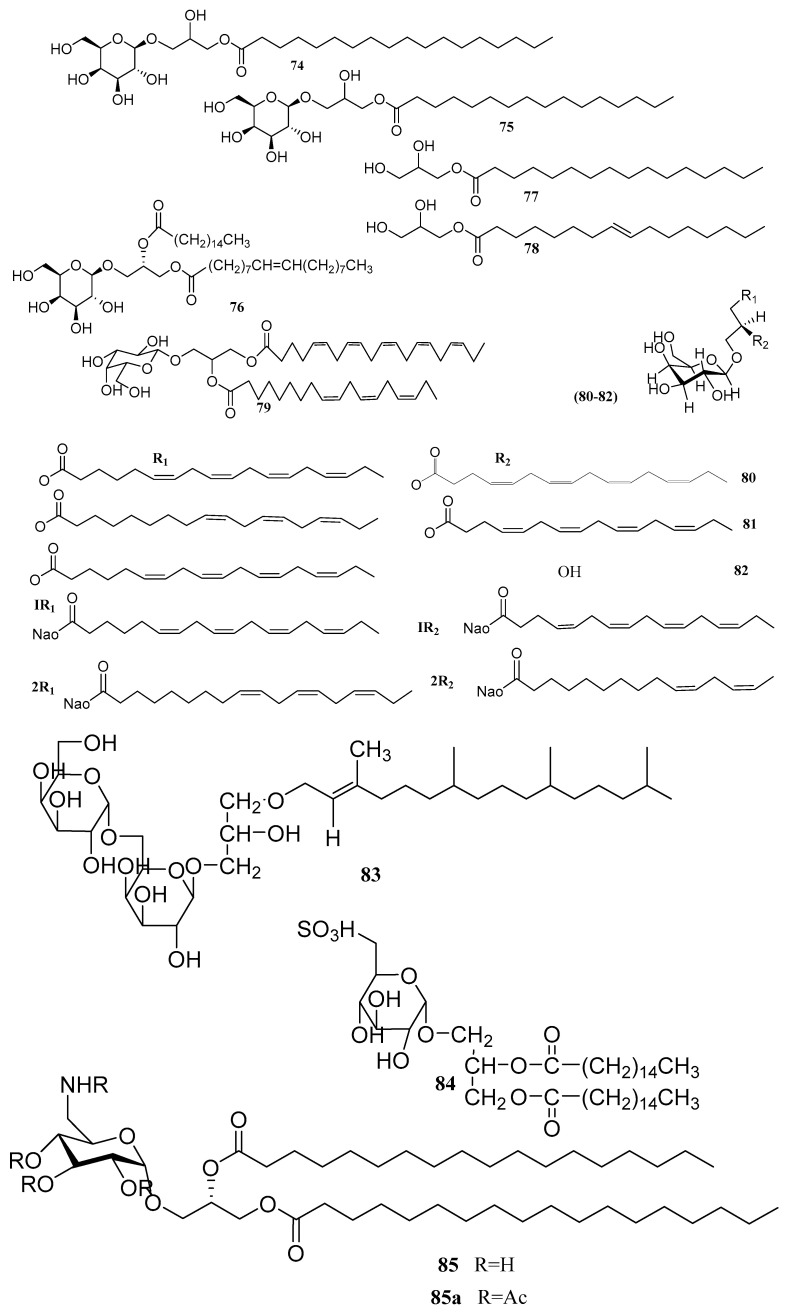
The names of compounds are represented as **74**–**85** in Table 6.

Sun [61] isolated three glycoglycerolipids: 1-*O*-octadecanoic acid-3-*O*-β-d-galactopyranosyl glycerol (**74**), 1-*O*-palmitoyl-3-*O*-β-d-galactopyranosyl glycerol (**75**) and 1-*O*-palmitoyl-2-Ooleoyl-3-*O*-β-d-galactopyranosyl glycerol (**76**). Two monoglycerides: glycerol monopalmitate (**77**) and 9-hexadecenoic acid,2,3-dihydroxypropyl ester (**78**), obtained from green algae *Ulva prolifera*, illustrated noteworthy antialgal activity against numerous red tide microalgae [61]. Moreover, a published study [59] reported “Monogalactosyl diacylglycerol”, 1-eicosapentaenoyl-2-linolenoyl-3-galactosylglycerol (**79**), from the *C. racemosa*, along with β-sitosterol (**2**), chlorophyll a (**3**), and unsaturated hydrocarbons from *Caulerpa racemosa*. Monogalactosyl diacylglycerols have also shown cytotoxic and anti-inflammatory activities in RAW 264.7 macrophage cells with IC_50_ values of 60.06 and 65.70 μg/mL, respectively. Zhe Fang [68] reported Capsofulvesins (**80**A–**82**C), (**80**, capsofulvesin A), (**81**, capsofulvesin B) and (**82**, capsofulvesin C) from green algae *Capsosiphon fulvescens*. The mentioned compounds presented IC_50_ values of 53.13 ± 2.83, 51.38 ± 0.90 and 82.54 ± 0.88 μM for determining the inhibitory activity of acetylcholinesterase (AChE), respectively, and IC_50_ values of >132.28, 114.75 ± 4.13 and 185.55 ± 6.95 μM for BChE assay. A novel compound, galactosyl glycerol-lipids (GGL) (**83**), consists of an α1, 6-galactobiose and glycerol backbone attached to an ether-linked phytol from the marine green alga *Ulva pertusa* [69,70]. Wang obtained a sulfoquinovosyl diacylglycerol (SQDG) compound (**84**) (that is not a sulfated polysaccharide) with antiviral activity [71,72] from the invasive alga *Caulerpa racemosa* emerging in the South China Sea. The SQDG compound had an outstanding antiviral activity against HSV-2, with an inhibitory concentration of 50 percent (IC_50_) 15.6 µg/mL against both normal and clinical HSV-2 strains, but only mild antiviral effects against HSV-1 and Cox B3. It was discovered a novel glycoglycerolipid presenting the extremely rare 6-deoxy-6-aminoglucose moiety avrainvilloside (**85**) obtained from the Dominican green alga *Avrainvillea* nigricans. Avrainvilloside (**85**) was inactive throughout preliminary cytotoxicity tests (IC50 > 10 µg/mL for WEHI 164 cells, murine fibrosarcoma) [73]. The chemical structures of glycerol and lipids from *Chlorophyta* are presented in Figure 9 and Table 6 describes the species and their bioactivity.

### 3.7. Polysaccharides (Ulvans) from Green Algae

Polysaccharides are monosaccharides polymers (sugars) connected by glycosidic (ether) links and constitute a structurally complex class of biological macromolecules. The structural complexity of these compounds stems from the several different sugars and sugar derivatives in polysaccharides, such as uronic acid, since each sugar can be covalently connected to other sugar by several different positions on a chain. They are used commonly as foods and as pharmaceuticals. Chlorophytes cell walls consist mainly of diverse and complex polysaccharides, so they cannot be accumulated here, ulvans, a polysaccharide being taken as another example.

The cell walls of green algae (Chlorophyte) are usually made up of polysaccharide, especially ulvans [74]. Ulvans are typically sulfated polysaccharides, consisting of a central framework of disaccharide modules, l-rhamnose 3-sulphate being linked to (i) ulvabiouronic acid unit A; (ii) ulvabiuronic acid unit B; (iii) ulvabiose unit A; or (iv) ulvabiose unit B [75,76]. However, the main repetitive disaccharide units mentioned in ulvan are ulvanobiouronic acid 3-sulfate composed of either glucuronic [1-4)-β-d-Glcp-(1-4)-α-l-Rhap3*S*-(1-3] n or iduronic [1-4)-α-l-Idop-(1-4)-α-l-Rhap3*S*-(1-3] n acid [74]. These polysaccharides are considered as relevant macromolecules that can be identified in the extracellular matrix, being crucial to mechanical, ionic and osmotic functions [77,78].

Chlorophyte genus, in particular, *Ulva* (family Ulvacae) has the ability to produce ulvans moieties of various sugar units that have special and numerous pharmacological applications. *Ulva pertusa*, isolated from heteropolysaccharides, was proven to show high inhibitory activities vs. hydroxyl and superoxide radicals. The low molecular weight, 28.2 kDa ulvan, also revealed strong reduction capacity and strong metal chelating properties, rendering it a powerful antioxidant agent [79,80]. An in vivo study highlighted the antioxidant capacity of *Ulva pertusa* sulfated Ulvan derivative in the liver of hyper-lipidemic rats. The high sulphate content improved the antioxidant activity and these polysaccharides were shown to protect liver tissue against a cholesterol-rich diet in rats and thus may be regarded as possessing antihyperlipidemia capabilities [81,82]. A high molecular weight (approximately 253 kDa) of *Ulva lactuca* sulfated polymer has been shown to inhibit a neurotropical flavivirus, namely the Japanese encephalitis virus (JEV), by obstructing its adsorption and cell penetration. It has also been shown to cause hind limb paralysis in a mice experiment [83]. Additionally, the *Ulva lactuca* polysaccharides extract can effectively protect the liver from damages due to abnormal mitochondrial development, lipid droplet deposits and hepatic protein thiols. In the case of an intoxicated rat model with d-galactosamine, this polymer may decrease LDL vitamin alleviate and also reduce glutathione showing antihyperlipidemic activity in rats [84,85].

## 4. Applications of Green Algae

### 4.1. Chlorophyte as a Spring of Pharmaceuticals and Nutraceuticals

Microalgae represent a diverse and interesting group of microscopic plants that encompasses a relatively high percentage of protein, ranging from 50 to 70% (50% in meat and 15–17% in wheat), 30% in lipids and 40% in glycerol, and a fairly average percentage of carotene (8–14%) and vitamins (B group vitamins, D, E, K, etc.). Microalgae present various physiological and biochemical properties in comparison with other plants and organisms [86]. Moreover, algal organisms represent a rich source of new primary and secondary metabolites biologically active [87]. These metabolites are potential bioactive compounds important for the pharmaceutical industry and may impact it. Seaweeds and their extracts may have various biological activities which include antitumor [88], anti-Alzheimer disease [89], antiprotozoal [90], antiviral [91], antioxidant [92] and cytotoxic action against cell lines in human cancer [14] and some seaweed extracts were also reported to exhibit antimicrobial activity [7,8]. The antimicrobial activity of the macroalgae was determined by the biologically active compounds also containing antibacterial properties, such as cycloeudesmol, lyengaroside A, meroditerpenoid, neoirietetraol, diterpene-benzoate, polybrominated indoles, halogenated sesquiterpene alcohol, lanosol enol ether, diterpene benzoic acids, callophycoic acids, halogenated diterpene-phenols, callophycols and eicosanoids [93]. Elnabris, in a 2013 publication, demonstrated that extracts of marine algae belonging to *Chlorophyta*, namely *Ulva lactuca* and *Enteromorpha compressa* were the most aggressive organisms, and *U. lactuca* proved also to be the most effective [94].

Carotenoids are an effective source of pigmentation; additionally, the antioxidant properties as well as their action in preventing cancer are beneficial for human health, at the skin level having an important role, protecting against UV radiation [95]. Undoubtedly, these compounds are utilized in pharmaceuticals and nutraceuticals. There was a significant increase in skin elasticity and more pronounced skin hydration based on the results of studies on these molecules used in cosmetics. Fucosterol shows strong antioxidant activity, among others, as a terpenoid isolated from *Ecklonia stolonifera*. Fucoxanthin can be an effective ultraviolet protector in cosmetics and sunscreen to delay photoaging. The seaweed tocopherol content was analyzed, revealing that microalgae tend to produce an essential amount of this compound. It is efficient for skin safety to be considered to have an important role in preventing skin and eye pathologies determined by light. Tocopherol is used as a food preservative in many consumer products, as sunscreen in cosmetics, etc. [62]. Carotenoids have a huge prospective for the cure of degenerative disorders, such as macular degeneration and eye cataract [96]. Ketocarotenoid astaxanthin is used in avoiding various human pathological processes like skin UV-mediated photooxidation, inflammation; prostate and mammary carcinogenesis, ulcers and age-related diseases [97]. Green algae have a huge chemical diversity and unique properties based on which they can be used in many ways, like antioxidants, in cosmetics, antibacterial, antiviral, anticancer, etc. Some are discussed below.

#### 4.1.1. Antioxidants

*Ulva fasciata* Deliles antioxidant properties for the sesquiterpenoids were discovered in green algae when using the free-radical-scavenging assays [98]. The *Ulva lactuca* is enormously acknowledged to have antioxidant properties in flavonoids [99,100]. Info from animal studies unveiled free-radical-scavenging effects of a reticulate hot water extract, due to which hepatic oxidative stress remained reduced [101]. The total content of phenol from the extract of *Cassytha filiformis filiformis* (39.31 ± 0.39 mg of AGE/g extract) was markedly higher (*p* < 0.05) by carrying out *Cassytha filiformis* antioxidant power ABTS (2,2′-azino-bis(3-ethylbenzothiazoline-6-sulfonic acid) and DPPH (2,2′-diphenylpicrylhydrazyl) assays. The extract of *C. filiformis* (IC50 = 3.49 ± 0.01 and 2.18 ± 0.02 mg/mL) was importantly greater (*p* < 0.05), so it is indorsed that the *C. filiformis’s* methanol extract is considered as a treasure of secondary metabolites with antioxidant properties [102].

#### 4.1.2. Cosmetics

Green algae are an offering source for cosmetic colorants, phenolic compounds, sterols, vitamins and other therapeutic agents [103]. Some of the bioactive agents (cosmetics) of isolated green algae compounds are used as skin moisturizing and protecting agents, creams with antistretch markings, body ointments, eye balms, face masks, antiaging washing gel, natural sunscreen, body scrubs, face peeling and face salves, firming body liniment, body unguents, purgative gels, fluids, stimulants, shampoos, day and/or night face cream, antielastase, collagen synthesis stimulation, chelating, inducement of collagen making via TGF-β, elastin, proliferation of collagen biosynthesis, anti-photo-aging agents, radical scavengers, colorants, cytoprotective Nrf2-ARE pathway, antiadhesive agents, antiwrinkling, emulsion stabilizers, immune-stimulants, potential for the treatment of histamine-related inflammatory illnesses including atopic dermatitis (AD), inhibition of hyaluronidase, antiallergic, hair growth, adipogenesis inhibitory effect, tyrosinase inhibitors, whitening agents, anticoagulant and topical cosmetic formulations to treat or avoid cellulite, augmented illumination for age spots and skin fall, UV emission defending and comforting, emollient, humectant, oral care, skin conditioning, antifungal and antiseptic, maintaining skin consistency and elasticity, antiacne makeup and many others uses [104].

#### 4.1.3. Antibacterial

A further relevant target of algal extracts comprises oral microbes. *Ulva linza* extracts have antibacterial agents against intermedia *Porphyromonas *gingivalis** and *P. intermedia*. Bioactive compounds (stearidonic acid) SA and (gamma-linolenic acid) GLA segregated from *Ulva linza* proved efficient antibacterial activity against *P. intermedia* and *Porphyromonas gingivalis* with MIC values of 39.06 µg/mL and 9.76 µg/mL, respectively [105]. *Ulva lactuca* showed an important antibacterial action against *Escherichia coli* while *Ascophyllum nodosum* was extra perceptive on *Micrococcus luteus* and *Brochothrix thermosphacta. Avrainvillea* sp. consists of bromophenols that pointed out inhibitory activity against both *Bacillus subtilis* and *Staphylococcus aureus*, which were also active against *Pseudomonas* *aeruginosa* as well as Escherichia coli, *Serratia marcesens* and *Candida albicans* [8]. *Utricularia rigida* composed of fatty acid obtained from Ghar el Melh lagoon can be used for the development of innovative antibacterial ingredients against human marine diseases [106].

#### 4.1.4. Anti-SARS-CoV-2 (COVID-19)

Currently, there is an urge of vaccine which might proceed to immunization against the deadly virus SARS-CoV-2 (COVID-19), but some traits of numerous sea weeds may provide an insight into possible remedies to this global pandemic in the near future. Several marine algae species possess large numbers of hierarchical sulfated polysaccharides which are proved to arrest the replication phase of enveloped viruses such as *Ulvans* [107] and *Caulerpa* [108]. A well-known alkaloidal compound caulerpin was studied In-silico for its antiviral activity against SARS-CoV-2 proteases as a monotherapy and also as a combination therapy with other predicted compounds having efficacy against SARS-CoV-2 such as chloroquine, hydro chloroquine, lopinavir and simeprevir. The results have revealed that caulerpin and its derivatives have shown high binding energies towards SARS-CoV-2 protein receptors as compared to all other predicted drugs. Furthermore, it has been revealed that, by adding different functional groups to the caulerpin structure like vinyl, halogen and NH_2_ enhanced the antiviral activity; as well, by adding alkyl groups, are decreased the binding affinities which in turn also decline antiviral activity. In addition, the molecular simulations data showed that the control drug simeprevir and caulerpin derivatives in combination have not shown any fluctuations in the protein and was stable, evidencing that caulerpin and its derivatives can be used as a combinational agent with other drugs, in order to destabilize the SARS-CoV-2 spikes protein [108].

#### 4.1.5. Anticancer

Dichloromethane methanol extracts are derived from two green algae named as *Udotea flabellum* and *U. Conglutinate* chlorophycean algae. The anticancer activity was observed on human melanoma cell line HeLa [109] comparing 22.5 vs. 22.2 µg/mL, respectively. It has been stated that *Udotea flabellum* extracts present antiproliferative activities of the cell lines HeLa, SiHa and KB [109]. *Enteromorpha intestinalis* and *Rhizoclonium riparium*s’ methanol extracts with IC50 values 309.048 ± 3.083 µg/mL and 506.081 ± 0.714 µg/mL were proved to be antiproliferative against cervical cancer cell line HeLa [110]. *Enteromopha prolifera* is another genus of green algae that had a suppressive activity with 51.7% for Erhlich’s carcinoma inhibition [111]. *Caulerpa*-isolated compounds were assayed against human cancer cell lines A 549 (human cancer carcinoma) and HL-60 (promyelocytic leukemia cells), among isolated compounds, α-tocopherol quinone showed restrained cytotoxicity towards HL-60 and low cytotoxicity towards A-549 [14,55]. Caulerpin’s IC50 values were recorded 20 µM against certain cancer cell lines (T47-D, MCF-7, MDA-MB-231, PC3 DU145, HMEC, HCT116, HT29, LOVO and SW480) but its mechanism of action disclosed that caulerpin obstructs hypoxia-inducible factor 1 (HIF-1) at 10 µM concentration and blocks the induction of HIF-1α protein, an essential oxygen-regulated subunit, under hypoxic circumstances. Caulerpin also has an effect on the migration of tumor cells when the concentration-dependent migration of metastatic MDA-MB-231 cells has been suppressed, with better results being noticed at 30 µM. Operation with Caulerpin in vivo combines with 3-bromopyruvate on xenografts implanted on an athymic nude mouse model carrying SW480. Combination therapy with caulerpin presented fabulous tumor regression. Additional inspection shows that in this combination therapy proliferating cell nuclear antigen (PCNA) and phosphorylated mammalian target phrase rapamycin (p-mTOR) are prevented, showing the important role of adenosine monophosphate-activated protein kinase (AMPK)/mTOR pathway in anticancer therapy. The cultivation of green algae is the best practice to increase the phenol and lipid content and thus improve SI on cancer cells, mostly on the SiHa and Hep-2 cell lines [112]. Through the P13K/Akt pathway, the hot water extract of *Capsosiphon fulvescens*’s polysaccharides determined apoptosis of gastric cancer cells, as well as dimethyl sulfoniopropionate and tertiary sulfonium metabolites, presented anticancer activity in mice with Ehrlich ascites carcinoma [113]. Nigricanosides A is a glycolipid obtained from *Avrainvillea nigricans*, being used in breast cancer cells in mitosis; sesquiterpenoid, known as Caulerpenyne from *Caulerpa taxifolia*, is used in colorectal cancer; sulfated polysaccharide, from *Ulva intestinalis*, is used in reducing tumor mass; sterol, from *Codium fragile*, is used in inducing apoptosis; glycoprotein from *Codium decorticatum* is used to induce apoptosis on MDA-MB-231 breast cancer cells [114]. A novel compound named as 25-hydroperoxy-6β-hydroxycholesta-4,23(E)-dien-3-one, extracted from *Galaxaura marginata*, displayed cytotoxicity against P338 (lymphocytic leukemia cells), A549 (human lung adenocarcinoma epithelial cell lines) and KB (KERATIN-forming tumor cell line HeLa). Cladophoropsis vaucheriaeformis shows tumorigenic behavior against murine lymphoid leukemia L1210 cells [115]. *Chaetomorpha compressa*, the marine green algae, has been proven to be a better anticancer agent against human breast carcinoma cells [116] and shows its antiapoptotic effects against human colon cancer cells HCT-116 [117] In short, green algae are the treasure of bioactive anticancer metabolites.

## 5. Algae as Nutraceuticals

### 5.1. Astaxanthin

The *Haematococcus pluvialis* is the key source of Astaxanthin, a powerful natural oxidant having more oxidative properties than vitamin C and E, β carotene, lutein, lycopene and zeaxanthin. Astaxanthin in the human diet can reduce inflammation, oxidative stress and improve the immune system of patients suffering from cardiovascular disease [118]. The involvement of the Natural Algae Astaxanthin Association (NAXA) has an important role in highlighting the advantages and value of astaxanthin. It points out the differences between natural algal astaxanthin and other synthetic sources. Astaxanthin consists of carbon precursors, it contains high lipid-soluble pigment, being basically an antioxidant with rather decreased activity but having good free radical terminator of each carotenoid. Astaxanthins are used as food supplements [119].

### 5.2. Omega 3 Polyunsaturated Fatty Acids

For the human’s body metabolism of PUFA is very important. Chlorophytes are used to extract these important and stable fatty acids. N-6 PUFA is a fatty acid abundant in food. The amount of fatty acids in algae makes them well-known bioactive compounds, very useful in the pharmaceutical industry. Some green algae medicinal applications of green algae are (1) *Enteromorpha*: can be used to treat hemorrhoids, parasitic disease, goiter, coughing and bronchitis; fever reduction capacity and ease pain; (2) *Corallina:* can be used as a pesticide and so on [119]. In short, chlorophytes are extensively used nutritionally as well as pharmaceutically because of their countless benefits. Some unsaturated fatty acids have vital therapeutic properties and benefits for the body. Omega-3 fatty acids lower cholesterol and fat levels in the bloodstream and “cleanse” the blood vessels lining [120]. The use of ω-3 fatty acids as a remedy for heart disease, coronary disease, inflammation, rheumatoid arthritis and immunodeficiency diseases has been revealed by some research [97,121].

## 6. Conclusions and Future Perspectives

Structural wise ultimate variety of natural products from green algae (*Chlorophyta*) is the endless effort of the researchers to move forward the green algae products into medical applications. However, the compounds of green algae have been over all ignored with many barricades, including the testing of crude extracts of green algae to neither isolate nor elucidate their structures to transmute the extract into pharmaceutical value [12]. Although these challenges hinder the biotransformation of biomolecules into clinical applications, alternative strategies can be amended to overcome these challenges such as large-scale collection and extraction to isolate more bioactive metabolites from green algae; most important, the full screening of chemically diverse bioactive compounds produced by algae during environmental stress is also claimed. To obtain larger amounts of such compounds, new molecular biology techniques (genomic studies for identification, metabolic engineering, hybrids, as well as other new effective extraction, purification processes) should be adapted to improve the production of these compounds.

Green algae are the treasure of protein, vitamins, pigments, carotenoids, fatty acids, polyphenol, etc., which neither solved nutraceutical related problems, but also made a path for pharmaceuticals. As there were discussed in this review, representatives of polyphenolic compounds can be used as antioxidant, antibacterial, antiviral, and inhibitory substances on tumor cell development (melanoma, lung and kidney carcinoma). Similarly, β-caroten, Astaxanthin, Lutein used as antioxidants, vitamin precursors, immune activators, anti-inflammatory, antihypertensive neuroprotective properties, effective action against cancer, atherosclerosis, ulcers and cardiovascular diseases, decrease the incidence of the metabolic syndrome, adiposity and serum triglyceride concentrations, strengthen the immune resistance to viral, bacterial, fungal and parasitic infections.

**Figure 10:**
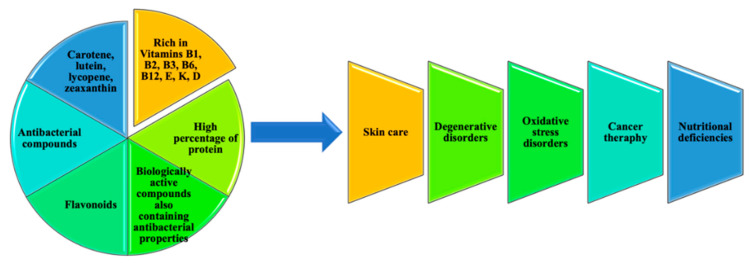
Properties and applications of green algae.

In this review, there have been described different classes of secondary metabolites related to chlorophytes of a different genus (in particular *Caulerpa, Ulva, Tydemania, Penicillus, Codium, Capsosiphon, Avrainvillea, Udotea* and *Chlorodesmis*). The main source still needs to be explored. Most of the compounds (especially alkaloids) repeatedly isolated from different species of green algae are of medical interest, having multiple pharmacological activities, summarized and schematically presented in Figure 10.

At present, PTP1B is being studied as a new therapeutic target class for cancer. In human clinical trials, no competitive small-molecule protein tyrosine phosphatase inhibitors are present or may be identified in small numbers. This review-type paper has presented Caulerpin as a potent candidate for treating cancer (by inhibiting the development of cancer cells); most interestingly, they also discussed its evident pharmacological action, motivating researchers to further focus on algae.

A lot of research has been conducted on the biological potential of crude extracts of these macroalgae, less focus being given to the isolation of active compounds that are responsible for these biological activities. Hence, the potential use of these chlorophytes is expected to lead to medicinal chemistry in the development of new bioactive compounds.

## Figures and Tables

**Table 1:** Alkaloids and prenylated compounds from *Chlorophyta*.

Compound	Species	Bioactivity	Ref.
Racemosin A (**1**)	*Caulerpa racemosa*	Neuroprotective	[26]
Racemosin B (**2**)	*Caulerpa racemosa*	Neuroprotective	[26]
Racemosin C (**3**)	*Caulerpa racemosa*	Significant PTP1B inhibitor	[27]
Caulerpin (**4**) Caulerpic acid (**5**)	*Caulerpa racemosa* and *Caulerpa genus*	Strong PTP1B inhibitor	[28]
Caulersin (**6**)	*Caulerpa serrulata*	PTP1B inhibitor	[30]
Caulerchlorin (**7**)	*Caulerpa racemose*	Weak antifungal	[31]
Caulerprenylols A (**8**)	*Caulerpa racemosa*	Antifungal	[26]
Caulerprenylols B (**9**)	*Caulerpa racemosa*	Antifungal	[26]
Pyrrolopipera-zine-2,5-dione (**10**)	*Ulva prolifera*	Antialgal	[40]
7-Hydroxycymo-pochromanone (PBQI) (**11**)	*Cymopolia barbata*	Chemotherapeutic	[41]
7-Hydroxycymo-polone (PBQ2) (**12**)	*Cymopolia barbata*	Chemotherapeutic, Anticancer colon cell	[41]
3′-methoxy-7-hydroxycymopolone (**13**)	*Cymopolia barbata*	Antimutagenic against *S typhimurium*	[41]
3-hydroxycymopolone (**14**)	*Cymopolia barbata*	Antimutagenic against *S typhimurium*	[41]
3,7-hydroxycymopolone (**15**)	*Cymopolia barbata*	Antimutagenic against *S typhimurium*	[41]
7-dihydroxycymo-pochromenol (**16**)	*Cymopolia barbata*	Antimutagenic against *S typhimurium*	[41]
Derivatives of cymopochromenol (**17**)	*Cymopolia barbata*	Antimutagenic- *S typhimurium*	[41]

**Table 2:** Sesquiterpene metabolites derived from *Chlorophyta*.

Compound	Species	Bioactivity	Ref.
Caulerpal A (**18**)	*Caulerpa taxifolia*	hPTP1B inhibitor	[21]
Caulerpal B (**19**)	*Caulerpa taxifolia*	hPTP1B inhibitor	[21]
Acetylene Sesquiterpenoid Esters (**20–28**)	*Caulerpa prolifera*	Antibacterial	[52]
Acetylene Sesquiterpenoid Esters (**29–34**)	*Caulerpa prolifera*	Antibacterial	[52]
Guai-2-en-10α-ol (**35**)	*Ulva fasciata*	Antibacterial	[53]
guai-2-en-10α-methanol (**36**)	*Ulva fasciata*	Antibacterial	[53]
Guai-2-en-10α-methyl methanoate (**37**)	*Ulva fasciata*	Antibacterial	[53]

**Table 3:** Diterpenoids metabolites derived from *Chlorophyta*.

Compound	Species	Bioactivity	Ref.
labda-14-ene-8-ol (**38**)	*Ulva fasciata*	Antibacterial	[54]
labda-14-ene-3α,8α-diol (**39**)	*Ulva fasciata*	Antibacterial	[54]
labda-14-ene-8α,9α-diol (**40**)	*Ulva fasciata*	Antibacterial	[54]
labda-14-ene-8α-hydroxy-3-one (**41**)	*Ulva fasciata*	Antibacterial	[54]
ent-labda-13(16),14-diene-2-one (**42**)	*Ulva fasciata*	Antibacterial	[54]
ent-labda-13(16),14-diene-3α-ol (**43**)	*Ulva fasciata*	Antibacterial	[54]
ent-labda-13(16),14-diene-3α-ol (**44**)	*Ulva fasciata*	Antibacterial	[54]
racemobutenolids A, B (**45ab**)	*Caulerpa racemosa*	-	[56]
4,5-dehydrodiodictyonema A (**46**)	*Caulerpa racemosa*	PTP1B inhibitor	[56]
an α-tocopheroid,α-tocoxylenoxy (**47**)	*Caulerpa racemosa*	PTP1B inhibitor	[56]

**Table 4:** Terpenoid metabolites derived from *Chlorophyta*.

Compound	Species	Bioactivity	Ref.
Lanosta-8-en-3,29-diol-23-oxo-3,29-disodium sulfate (**48**)	*Tydemania expeditionis*	cytotoxic tumor cell	[57]
Capisterones A (**49**)	*Penicillus capitatus*	potent antifungal	[58]
Capisterones B (**50**)	*Penicillus capitatus*	potent antifungal	[58]
Squalene (**51**)	*C. Racemose*	-	[59]
Dwarkenoic acid (**52**)	*Codium dwarkense*	alpha-glucosidase inhibitor	[60]
Loliolide (**53**)	*Ulva prolifera*	-	[61]
Lsololiolide (**54**)	*Ulva prolifera*	-	[61]

**Table 5:** Steroids and fatty acid metabolites derived from *Chlorophyta*.

Compound	Species	Bioactivity	Ref.
Cholest-5-en-3-ol (**55**)	*Ulva prolifera*	Antialgal	[63]
(8*E*,12*Z*,15*Z*)-10-hydroxy-8,12,15-octadecatrien-4,6-diynoic acid (**56**)	*Caulerpa racemosa*	-	[64]
Hexadeca-4,7,10,13-tetraenoic acid (HDTA) (**57**)	*Ulva fasciata*	Algicidal	[63]
Octadeca-6,9,12,15-tetraenoic acid (ODTA) (**58**)	*Ulva fasciata*	Algicidal	[59]
α-linolenic acid (**59**)	*Ulva fasciata*	Algicidal	[59]
β-sitosterol (**60**)	*Caulerpa racemosa*	-	[59]
(1, iyengadione) (**61**)	*Codium iyengarii*	-	[65]
iyengaroside-A (**62**) and B (**63**)	*Codium iyengarii*	Antibacterial	[65]
Clerosterol galactoside (**64**)	*Codium iyengarii*	-	[65]
3-hydroxy-octadeca-4(*E*),6(*Z*),15(*Z*)-trienoic acid (**65**)	*Tydemania expeditionis*	Antitumor	[57]
3-hydroxyhexadeca-4(*E*),6(*Z*)-dienoic acid (**66**)	*Tydemania expeditionis*	Antitumor	[57]
(24*R*)-5,28-stigmastadiene-3β,24-diol-7-one (**67**)	*Ulva australis*	Aldose reductase inhibitor	[66]
(24*S*)-5,28-stigmastadiene-3β,24-diol-7-one (**68**)	*Ulva australis*	Aldose reductase inhibitor	[66]
24*R* and 24*S*-vinylcholesta-3β,5α,6β,24-tetraol (**69**)	*Ulva australis*	Aldose reductase inhibitor	[66]
keto-type fatty acid (**70**)	*Ulva lactuca*	ARE activators	[67]
Shorter chain C16 acid (**71**)	*Ulva lactuca*	ARE activators	[67]
Amide derivative (**72**)	*Ulva lactuca*	ARE activators	[67]
(23*E*)-3b-hydroxy-stigmasta-5,23 dien-28-one (**73**)	*Caulerpa racemosa*	PTP1B inhibitor	[56]

**Table 6:** Glycerol and lipids metabolites derived from *Chlorophyta*.

Compound	Species	Bioactivity	Ref.
1-*O*-octadecanoic acid-3-*O*-β-d-galactopyranosyl glycerol (**74**)	*Ulva prolifera*	Antialgal	[61]
1-*O*-palmitoyl-3-*O*-β-d-galactopyranosyl glycerol (**75**)	*Ulva prolifera*	Antialgal	[61]
1-*O*-palmitoyl-2-Ooleoyl-3-*O*-β-d-galactopyranosyl glycerol (**76**)	*Ulva prolifera*	Antialgal	[61]
Monopalmitate (**77**)	*Ulva prolifera*	Antialgal	[61]
9-hexadecenoic acid, 2,3-dihydroxypropyl ester (**78**)	*Ulva prolifera*	Antialgal	[61]
1-eicosapentaenoyl-2-linolenoyl-3-galacto-sylglycerol (**79**)	*C. racemosa*	Anti-inflammatory	[59]
Capsofulvesins (**80A–82C**)	*Capsosiphon fulvescens*	Acetylcholinesterase (ache) inhibitor	[68]
Galactosylglycerolipid (GGL) (**83**)	*Ulva pertusa*	-	[69,70]
Sulfoquinovosyl diacylglycerol (SQDG) (**84**)	*Caulerpa racemosa*	Antiviral against HSV-2	[71]
Avrainvilloside (**85**)	*Avrainvillea nigricans*	Inactive cytotoxic	[73]
